# The prognostic significance of high/positive expression of cyclin B1 in patients with three common digestive cancers: a systematic review and meta-analysis

**DOI:** 10.18632/oncotarget.21273

**Published:** 2017-09-26

**Authors:** Yong Wen, Lei Cao, Wen-Ping Lian, Guo-Xia Li

**Affiliations:** ^1^ Chinese Medicine Department, Southwest Medical University Affiliated Hospital, Luzhou 646000, China; ^2^ Department of Pediatrics, Gansu Provincial Maternity and Child Care Hospital, Lanzhou 730000, China; ^3^ Department of Clinical Laboratory, The Third People's Hospital of He’nan Province, Zhengzhou 450000, China; ^4^ Department of Pathology, Shanghai Minhang District Central Hospital, Shanghai 201199, China

**Keywords:** cyclin B1, gastric cancer, colorectal cancer, esophageal cancer, prognosis

## Abstract

Recently, several studies have reported that the expression of cyclin B1 may be associated with the prognosis of cancer. Nevertheless, their conclusions were still controversial. The present was designed to analyze and evaluate the prognostic role of cyclin B1 expression in patients with digestive cancer. PubMed, Embase, Cochrane Library and Web of Science were searched to January, 2017. Pooled odds ratio (OR) with 95% confidence intervals (CIs) were estimated. For the pooled OR estimates of OS, we performed subgroup analysis. Besides, sensitivity analysis was performed to examine the stability of the combined results. All statistical analyses were performed using standard statistical procedures provided in RevMan 5.2. A total of 12 studies (*N* = 2080 participants) were included for this meta-analysis. The positive/high expression of cyclin B1 had an obvious association with both 3-year overall survival (OR 0.21, 95% CI 0.12–0.37; *P* < 0.00001) and 5-year overall survival (OR 0.20, 95% CI 0.12–0.34; *P* < 0.00001) in esophageal cancer, and 5-year overall survival of colorectal cancer (OR 2.01, 95% CI 1.32–3.08; *P* = 0.001). This meta-analysis indicated that positive/high expression of cyclin B1 may have a close association with worse survival in patients with esophageal cancer, but better prognosis in patients with colorectal cancer.

## INTRODUCTION

Digestive malignancies, especially gastric cancer, colorectal cancer and esophageal cancer, are rampant in several countries around the world. Colorectal cancer, as one of the most common digestive malignancies, is occupying the fourth in diagnosed cancer and in the second place of leading death of patients with cancer in the United States [1]. In 2015, it is estimated that about five thousands of people will die of CRC [1, 2]. Colorectal cancer was reported the third most frequently found malignancy, with male incidence of percentage 12.3 and female 13.1% [3, 4]. Additionally, in spite of the early detection with endoscopic screening in North America, CRC is in the third places in most common cause of cancer-related deaths, including both of patients who underwent operation or not [5].

At present, several advances have been made in the treatment approaches, imaging techniques and staging procedures. However, in spite of advanced instruments like electronic gastroscope and electron colonoscopy which could early detect digestive lesion, there were still approximately 20% of patients finding to be metastatic when they were diagnosed [6, 7]. Therefore, further appropriate prognostic bio-markers are required to predict the prognosis of patients who underwent operation and their clinical status of tumor, then to guide the doctor to give more attention to patient who may experience poor prognosis.

In recent years, increasing number of studies have reported that the expression of cyclin B1 may be correlated with the poor outcome of patients with various cancers, including breast carcinoma [8], prostatic cancer [9], pancreatic malignancy [10], lung carcinoma [11], laryngeal cancer [12] as well as hepatocellular cancer [13], gastric cancer (GC) [14–17], colorectal cancer [18–21] and esophageal cancer (EC) [22–25]. However, the conclusions of these studies was still incongruent, for some studies drawing unsupported conclusions [18, 19]. Therefore, the present meta-analysis on the basis of relevant studies were conducted to analyze and assess the prognostic value and clinical significance of high or positive expression of cyclin B1, as well as its association with characteristics, in patients with three main digestive cancers, including gastric cancer, colorectal cancer and esophageal cancer.

## RESULTS

### Included studies and characteristics

**Figure 1:**
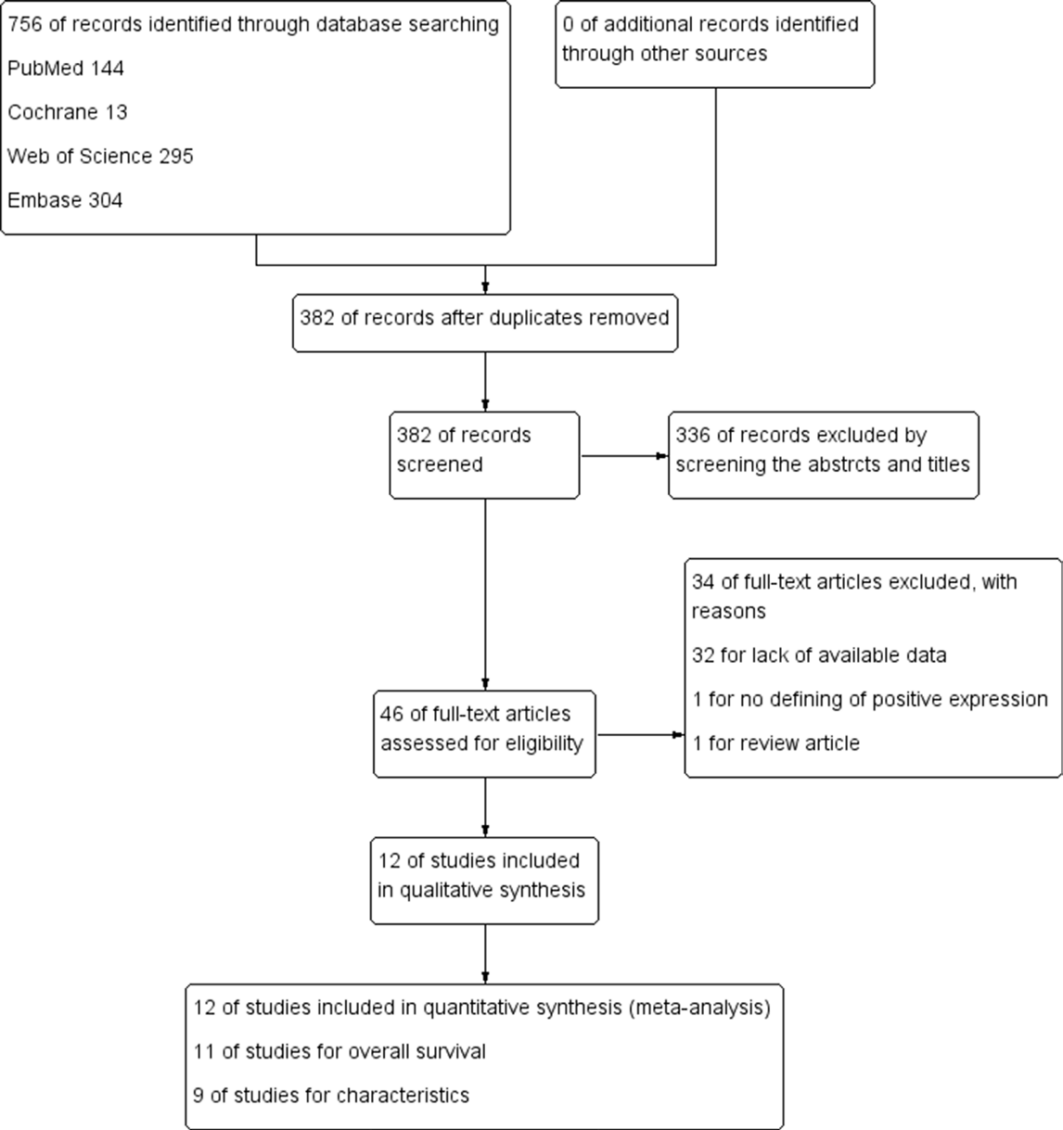
Flow diagram following the PRISMA template of the search strategy for expression of cyclin B1 in patients with cancer

After 34 studies were excluded according to our including criteria, eventually a total of 12 articles (2080 patients) were included for analysis and evaluation, of which 11 articles [14–20, 22–25] including 1971 patients were included for analyzing the correlation of positive/high expression of cyclin B1 and OS, 9 studies [15–17, 19, 21–25] including 1253 participants for analyzing the correlation between cyclin B1 and clinicopathological features (Figure 1). The excluding reasons were as follows: 32 studies for lack of available data; 1 study [26] for the definition of positive/high expression did not meet our including criteria; 1 study [27] was just a review article with no available data. Of the included studies, six were conducted in Japan, three in China, each one in Brazil, Germany and Korea. The sample sizes ranged from 23 to 482 patients. Each four studies were about gastric, colorectal and esophageal cancer. Patients in nine studies were treated with surgery, two treated with adjuvant therapy like chemotherapy, radiotherapy or chemo-radiotherapy, apart from surgery (Table 1).

**Table 1:** The characteristics of included studies

Studies	Country	No. of Patients	Tumor Location	Treatment	FT (month)	No. of Cyclin B1 (+/−)	Definition of positive/high expression	Clinical stage	P value	NOS score
Begnami et al. 2010	Brazil	482	GC	Surgery	28.3 (0.6–108.6)	229/231	IHC stained cytoplasm of the tumor cell > 10%	NR	‒	6
Fang et al. 2015	China	150	CRC	Surgery	NR	88/62	expression ratio > 3.33	I–IV	NR	7
Grabsch et al. 2004	Germany	342	CRC	Surgery, CT, RT or CRT	50.4 (5–136.8)	261/69	IHC cytoplasmic or nuclear staining > 10%	I–IV	0.5377	8
Huang et al. 2014	China	105	EC	Surgery	NR	28/17	IHC staining intensity and cell positive rate score 5–6/7,for ++/+++	I–III	0.01	6
Kim et al. 2007	Korea	23	GC	NR	68 (3–108)	20/3	IHC stained tumor cell > 5%	I–IV	NR	6
Korenaga et al. 2002	Japan	109	CRC	Surgery	NR	62/47	IHC stained either the nucleus or cytoplasm	NR	‒	6
Li JQ et al. 2003	Japan	194	CRC	Surgery	NR	68/75	IHC cytoplasmic or nuclear staining ≥ 4.6%	I–IV	0.119	8
Li YZ et al. 2009	China	336	GC	Surgery	45.4	222/114	IHC cytoplasmic or nuclear staining > 10%	I–IV	< 0.05	7
Murakami et al. 1999	Japan	87	EC	Surgery, CT	NR	63/24	IHC cytoplasmic staining > 5%	I–IV	> 0.05	6
Nozoe et al. 2002	Japan	120	EC	Surgery	NR	68/52	IHC nuclear and/or cytoplasmic staining > 10%	I–III	0.177	7
Takeno et al. 2002	Japan	71	EC	Surgery	NR	35/36	IHC staining > 20%	I–IV	< 0.0001	7
Yasuda et al. 2002	Japan	61	GC	Surgery	25.8	32/29	IHC cytoplasmic staining > 10%	I–IV	0.02	8

CRC, colorectal cancer; EC, esophageal cancer; GC, gastric cancer; FT, follow-up time (median and range); CT, chemotherapy; RT, radiotherapy; CRT, chemo-radiotherapy; NOS, Newcastle-Ottawa Scale; IHC, immunohistochemistry; NR, not report.

### The prognostic value of positive/high expression of cyclin B1

### The correlation between cyclin B1 expression and 3-year OS

**Figure 2:**
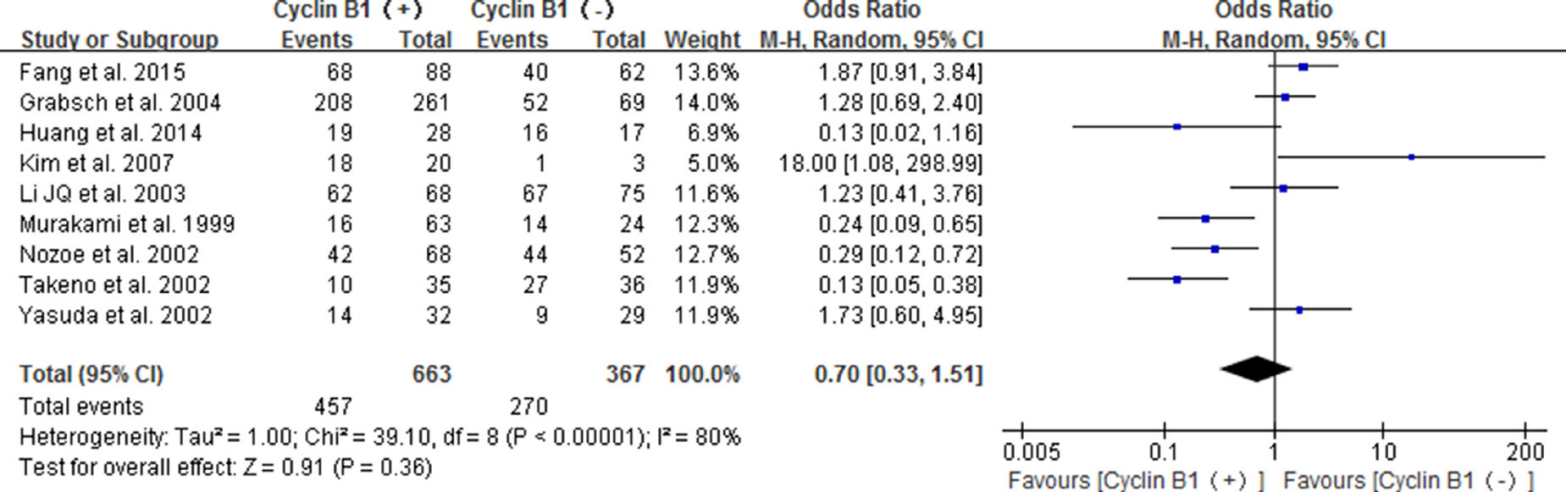
Forest plot for the association between positive/high expression of cyclin B1 and 3-year overall survival (OS) of overall types of cancer

A total of 9 studies [16–20, 22–25] were included to analyze the relationship of positive/high cyclin B1 expression with 3-year overall survival in patients with digestive cancer. The pooled results showed no significance for positive/high expression of cyclin B1 predicting the 3-year overall survival of patients(OR 0.70; 95% CI 0.33–1.51; *P* = 0.36) (Figure 2).

### The correlation between cyclin B1 expression and 5-year OS

**Figure 3:**
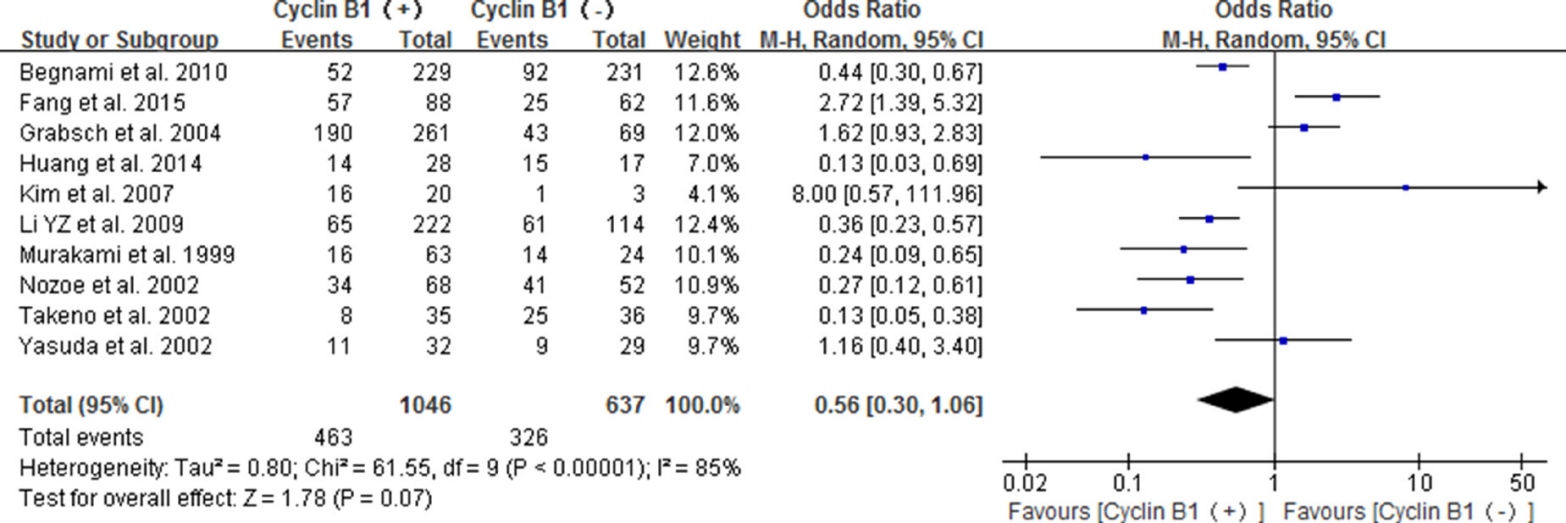
Forest plot for the association between positive/high expression of cyclin B1 and 5-year overall survival (OS) of overall types of cancer

Ten studies reported the association between positive/high expression of cyclin B1 and 5-year OS, of which four were about patients with esophageal cancer [22–25], four about gastric cancer [14–17] and two about colorectal cancer [18, 19]. The combined result indicated that the high/positive expression of cyclin B1did not significant correlate with 5-year OS of digestive cancer patients (OR 0.56, 95% CI 0.30–1.06; *P* = 0.07) (Figure 3).

### The correlation between cyclin B1 expression and 3-year OS of EC, GC and CRC

**Figure 4:**
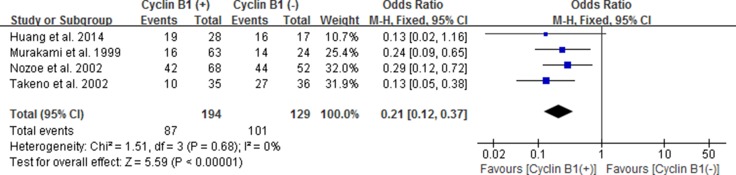
Forest plot for the association between positive/high expression of cyclin B1 and 3-year overall survival (OS) of esophageal cancer

Considering no significance in overall types of the three digestive cancers, we performed subgroup analysis according to gastric carcinoma (GC), colorectal carcinoma (CRC) and esophageal carcinoma (EC). The positive/high expression of cyclin B1 had an obvious association with 3-year OS in patients with esophageal cancer according to the pooled results (OR 0.21, 95% CI 0.12–0.37; *P* < 0.00001) (Figure 4). It revealed that esophageal cancer patients with positive/high expression of cyclin B1 had worse prognosis. However, no significant association between cyclin B1 and prognosis of patients with gastric cancer and colorectal cancer was found in our analysis.

### The correlation between cyclin B1 expression and 5-year OS of EC, GC and CRC

**Figure 5:**
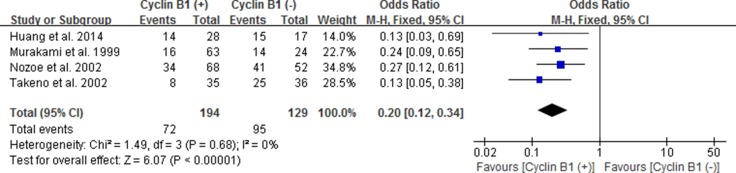
Forest plot for the association between positive/high expression of cyclin B1 and 5-year overall survival (OS) of esophageal cancer

**Figure 6:**
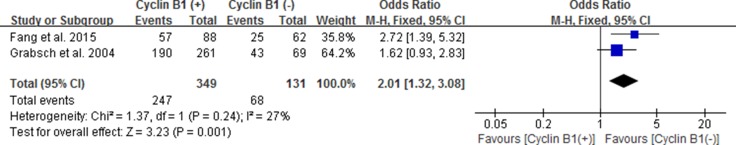
Forest plot for the association between positive/high expression of cyclin B1 and 5-year overall survival (OS) of colorectal cancer

Likewise, we also estimated the correlation between cyclin B1 expression and 5-year OS of esophageal carcinoma (EC), gastric carcinoma (GC) and colorectal carcinoma (CRC) by subgroup analysis. We used fixed effect models because of no significant heterogeneity between studies. Our results showed statistically significant correlation between positive/high expression of cyclin B1 and 5-year OS of patients with EC and CRC, with the pooled OR being 0.20 [95% CI 0.12–0.34; *P* < 0.00001] and 2.01 [95% CI 1.32–3.08; *P* = 0.001], respectively (Figures 5 and 6). However, interestingly, patients with positive/high expression of cyclin B1 had worse prognosis in patients with esophageal carcinoma, but better prognosis in patients with colorectal carcinoma. No significant association between cyclin B1 and prognosis of patients with gastric cancer was found in our analysis.

### The correlation between Cyclin B1 expression and clinicopathologic features

In order to explicate the point if the expression of cyclin B1 was correlated with clinical characteristics of patients, we explored whether Age, Gender, Size of tumor, Blood vessel invasion, Histologic type, Pathologic T, Pathologic N, Distant metastasis, Lymphatic vessel invasion and Clinical stage were correlated with high expression of cyclin B1. The results indicated significant association between positive/high cyclin B1 expression and Size of tumor (OR 0.60, 95% CI 0.38, 0.93; *P* = 0.02) and Distant metastasis (OR 0.30, 95% CI 0.13, 0.71; *P* = 0.006), using fixed-effect models. However, no significant association in other characteristics was found (Table 2).

**Table 2:** The association between Cyclin B1 and clinical features

Clinical features	No. of studies	No. of patients	Pooled results			Heterogeneity		
			OR	95% CI	*P* value	I^2^	*P* value	Analytical effect model
Age	5	1090	0.72	0.50, 1.02	0.07	0%	0.41	FEM
Gender	6	817	1.03	0.67, 1.57	0.91	0%	0.73	FEM
Size of tumor	3	832	0.60	0.38, 0.93	0.02	0%	0.73	FEM
Histologic type								
Well vs. Moderate	6	669	1.25	0.80, 1.93	0.32	0%	0.49	FEM
Well vs. Poor	6	812	1.40	0.44, 4.44	0.57	75%	0.001	REM
Moderate vs. Poor	6	1011	0.85	0.43, 1.69	0.64	58%	0.04	REM
Pathologic T	7	1402	0.74	0.41, 1.34	0.32	68%	0.005	REM
Pathologic N	8	1413	0.71	0.40, 1.25	0.23	69%	0.002	REM
Metastasis	4	807	0.30	0.13, 0.71	0.006	32%	0.22	FEM
Blood vessel invasion	6	1315	0.90	0.43, 1.86	0.77	70%	0.006	REM
Lymphatic vessel invasion	6	1319	0.85	0.43, 1.67	0.64	73%	0.002	REM
TNM stage	7	1802	0.67	0.29, 1.54	0.34	86%	< 0.00001	REM

REM, random-effect model; FEM, fixed effects model; OR, odds ratio; CI, confidence intervals.

### Subgroup, sensitivity analysis and publication bias

Subgroup analysis was conducted to find the prognostic effects of positive/high cyclin B1 expression on 3 and 5-year OS. Statistically significant effect of cyclin B1 expression on 3-year OS was observed in patients with stage I–III (OR 0.25; 95% CI 0.11, 0.58; *P*=0.001). For the effect of cyclin B1 expression on 5-year OS, significant results were found in subgroups of I–III (OR 0.23; 95% CI 0.11, 0.48; *P* < 0.0001) and TNM stage *P* < 0.05 (OR 0.32; 95% CI 0.13, 0.76; *P* = 0.010). In addition, there was good homogeneity among the studies in the subgroup of stage I–III for no significant heterogeneity was found, thus FEM was used to evaluate the pooled effects. No significant association was found in the subgroups of stage I–IV, TNM stage *P* < 0.05 and Cut-off value = 10% or ≤ 5% (Table 3).

**Table 3:** The pooled results of subgroups for the association between Cyclin B1 and 3-year OS or 5-year OS

Subgroups	Number of studies	Pooled results (3-year OS)			Pooled results (5-year OS)		
		OR	95% CI	*P* value	OR	95% CI	*P* value
Clinical stage							
I–IV	7	0.93	0.40, 2.17	0.86	0.77	0.32, 1.88	0.56
I–III	2	0.25	0.11, 0.58	0.001	0.23	0.11, 0.48	< 0.0001
*P* value of TNM stage							
< 0.05	4	0.34	0.05, 2.30	0.27	0.32	0.13, 0.76	0.010
> 0.05	4	0.59	0.24, 1.46	0.25	0.49	0.13, 1.91	0.30
Cut-off value							
10%	5	0.87	0.31, 2.41	0.78	0.59	0.31, 1.12	0.11
≤ 5%	4	1.25	0.35, 4.51	0.73	1.43	0.20, 10.38	0.72

OS, overall survival; OR, odds ratio; CI, confidence intervals.

We performed sensitivity analysis to examine the stability of the combined results of overall survival and to identify the source of heterogeneity by omitting any single study. Sensitivity analysis showed that the combined OR of 3-year OS of all types of cancer had high stability by omitting each single study, for that the pooled ORs ranged from 0.60 [95% CI 0.26, 1.39] after omitting the study of Fang et al. 2015 [18] to 0.62 [95% CI 0.27, 1.45] after omitting the study of Yasuda et al. 2002 [17]. However, for 5-year OS of all types of cancer, three particular studies, Fang et al. 2015 [18], Grabsch et al. 2004 [19] and Kim et al. 2007 [16], significantly affected the pooled results. The pooled ORs respectively changed from 0.56 [95% CI 0.30, 1.06] to 0.45 [95% CI 0.25, 0.81], 0.48 [95% CI 0.25, 0.93] and 0.50 [95% CI 0.26, 0.95] after excluding each of them from the meta-analysis seriatim. The pooled results of 3 and 5-year OS of esophageal cancer and colorectal cancer had high stability as well. No single study was found significantly affecting heterogeneity between studies.

**Figure 7:**
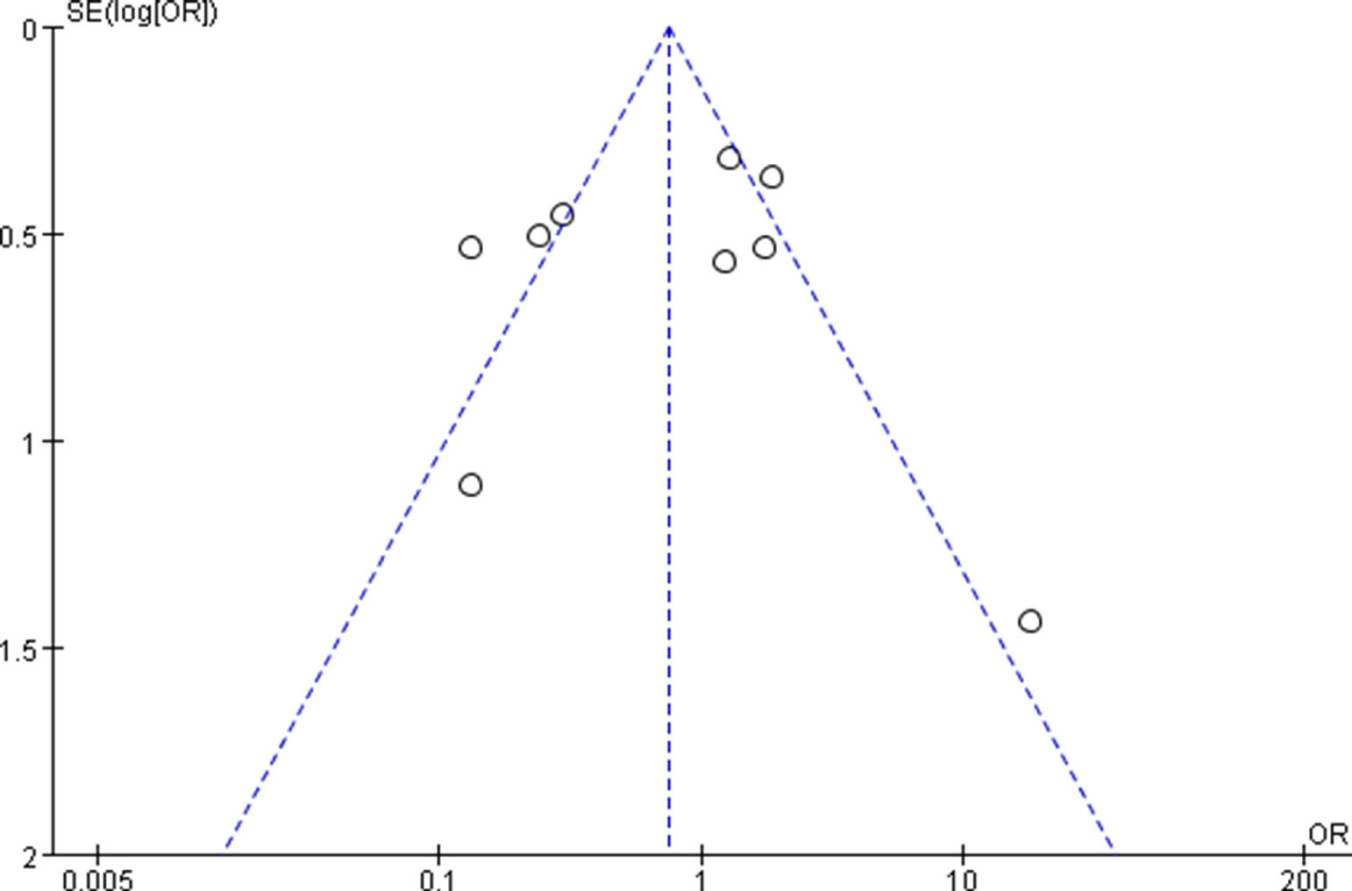
Funnel plots for detecting publication bias of the association between positive/high expression of cyclin B1 and 3-year overall survival (OS) of overall types of cancer

**Figure 8:**
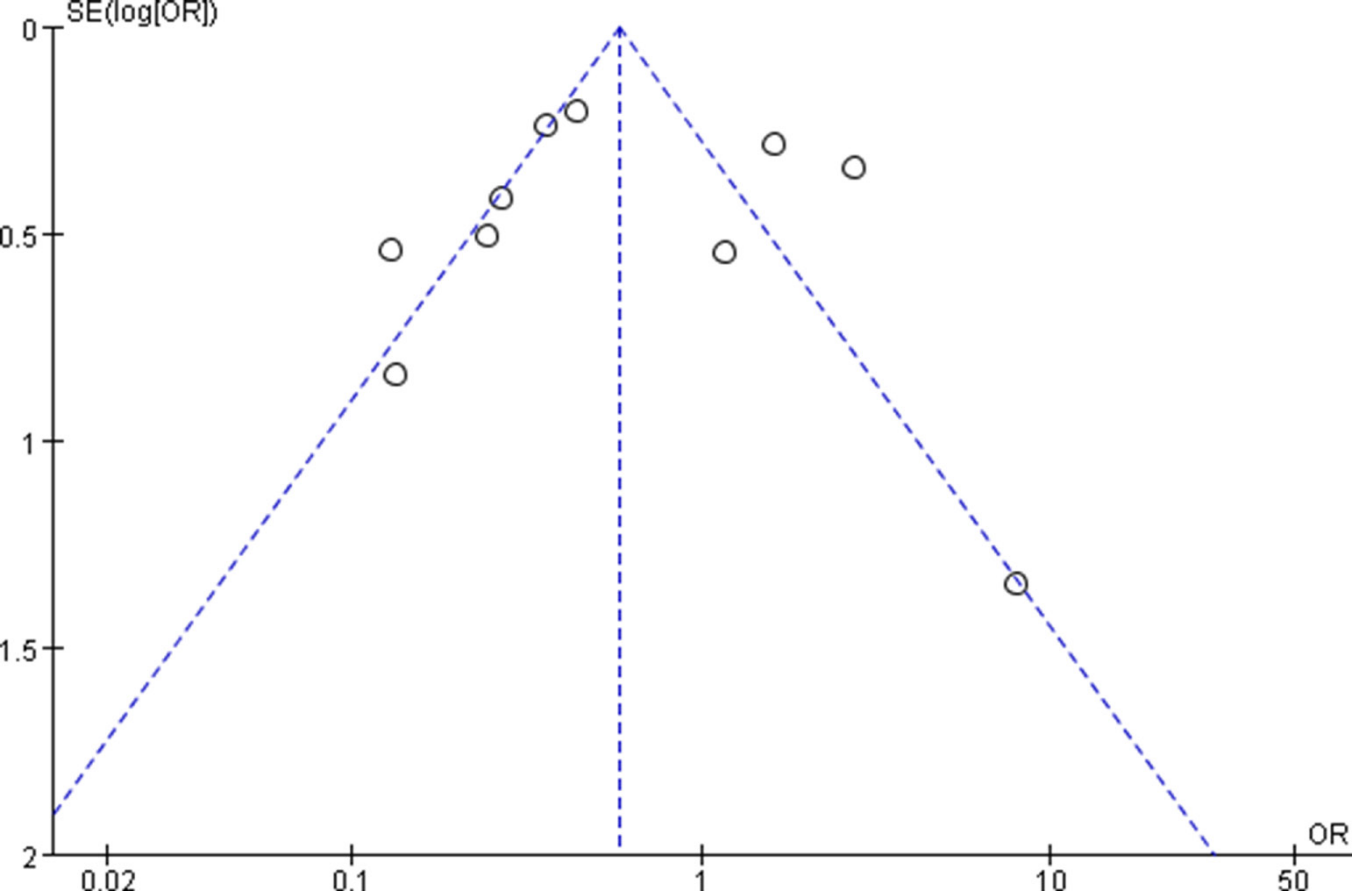
Funnel plots for detecting publication bias of the association between positive/high expression of cyclin B1 and 5-year overall survival (OS) of overall types of cancer

The funnel plots showed no obvious evidence of publication bias with regard to the effects on 3 and 5-year OS (Figures 7 and 8).

## DISCUSSION AND CONCLUSIONS

It has been implicated that high expression of cyclin B1, to a certain extent, may impede the autogenous controlling of cell growth and lead to a malignant phenotype [28]. The adjustion of cell cycle progression is strongly necessary for cells to keep gene integration which is very critical for cell survival. According to current research, cyclin B1 is one of a family of proteins varying in their expression levels during the cell proliferation cycle [29–32]. Cyclin B1 has various roles in cell proliferation cycle, including cell differentiation, cell apoptosis, and the (tumor cell) distant metastasis [33–37]. Increasing proofs have indicated that the important protein was high or over expressed in laryngeal cancer [12], prostate cancer [9], pancreatic carcinoma [10], lung carcinoma [11], breast carcinoma [8], hepatocellular cancer [13], esophageal cancer, gastric cancer and colorectal cancer [15, 20, 23, 24]. Murakami H et al. reported that the cyclin B1 prevalently expressed in patients with tumor deeper invasion than superficial invasion [25]. Song Y.M et al. suggested that the protein result in tumor distant metastasis likely via accelerate a transition of epithelial to mesenchyma [29]. The study from Wang A. et al. showed much higher expression of the protein in human colorectal carcinomas than did the non-neoplastic mucosa [26]. Besides, previous studies have also compared the different significance of positive cyclin B1 expression in nuclear and cytoplasm, and testified that the protein expression, especially nuclear dominant expression, can be significant as a prognostic indicator in esophageal cancer [24]. However, the detailed role and mechanism of cyclin B1 in prognosis of cancer patients was not clear. What's more, their results still remained inconsistent. The findings from Fang Y. et al. suggested that cyclin B1 could control the invasion and distant metastasis of colorectal cancer cells [18], which was opposite to with other studies [14–16, 20]. Besides, according to Grabsch's results, though this protein was frequently and early expression in malignancies, it did not predict the prognosis and recurrence risk for patients [19]. Considering all kinds of discrepancies above, further study was needed to unitize the different voice.

We perform the present analysis to demonstrate the significance of cyclin B1 expression for the survival of patients with digestive malignancies. Exceptionally, results of the present analysis did not remarkable support the consequence that high or elevated cyclin B1 expression was significantly correlated with the prognosis in all types of digestive cancer. However, surprisingly, we found the prominent pertinence between the high/over-expression of cyclin B1 and esophageal cancer or colorectal cancer. In esophageal cancer, cyclin B1 positive/high expression had an evident association with both 3-year OS (OR 0.21, 95% CI 0.12–0.37; *P* < 0.00001) (Figure 4) and 5-year OS (OR 0.20, 95% CI 0.12–0.34; *P* < 0.00001) (Figure 5). The pooled OR was 2.01 [95% CI 1.32–3.08; *P*=0.001] in colorectal cancer (Figure 6). Interestingly, though the significance was found both in esophageal cancer and colorectal cancer, it was a contrary clinical result. According to the results, patients with cyclin B1 positive/high expression experienced a poor prognosis and low 3 or 5-year OS in esophageal cancer, but high 5-year OS in patients with colorectal cancer, which was consistent with the study of Korenaga D et al. 2002 [21]. Ye C et al. [27] came to the same conclusion in esophageal carcinoma and colonic or rectal carcinoma. The reason for this inconsistent results may be the different expression modes of cyclin B1 in different tissue types of tumor. In addition, we also estimated association between elevated cyclin B1 expression and clinicopathological features. In the overall pooled analysis, the results suggested that cyclin B1 positive/high expression was statistically correlated with size of tumor lesions (OR, 0.60; 95% CI, 0.38, 0.93) and distant Metastasis (OR, 0.30; 95% CI, 0.13, 0.71), but not with other clinicopathological features such as gender, grade, Pathologic T, Pathologic N, Blood vessel invasion and Histologic type in the patients with digestive cancer. According to the study of Yasuda M et al. 2002, tumors tissue in which cyclin B1 expressing were prevalent in gerontal patients, in better differentiation and swelling-growth tumors, but was lower expression in poorly differentiated and invasive-growth tumor. The subgroup analysis they performed in study indicated that tumor histological type was one of remarkable factors relating to cyclin B1 protein expression [17]. In subgroup analysis of 3 and 5-year OS, the results indicated that positive/high cyclin B1 expression was prominently correlated with 3-year OS of patients with stage I–III (OR 0.25; 95% CI 0.11, 0.58; *P* = 0.001) and 5-year OS of patients with stage I–III (OR 0.23; 95% CI 0.11, 0.48; *P* < 0.0001) and TNM stage *P* < 0.05 (OR 0.32; 95% CI 0.13, 0.76; *P*=0.010), but not in other subgroups. According to the results from sensitivity analysis, we found that the study from Fang et al. 2015, Grabsch et al. 2004 and Kim et al. 2007 [16, 18, 19] had significant impacts on the pooled results. When the studies above was got rid from the analysis lists, the pooled OR became 0.45 [95% CI 0.25, 0.81], 0.48 [95% CI 0.25, 0.93] and 0.50 [95% CI 0.26, 0.95] for 5-year OS.

However, several limitations existed in thsi meta-analysis. One of the main deficiencies was the inconformity of the cut-off values (definition of positive/high expression of cyclin B1). The cut-off values ranged from 4.6% to 20%, but the majority of studies used 10%. The cut-off values may directly affect the results of analysis. In the study of Huang TY et al. 2014 [22], they classified the −/+ to negative, ++/+++ to positive to generate the survival curves, but Murakami H et al. regarded 1+ and 2+ as positive [25]. Besides, Takeno S et al. 2002 [23] suggested that elevated cyclin B1 expression was correlated remarkablely with a poor prognosis in patients with esophageal cancer when the cut-off value for positivity was set at 20% (*P* < 0.0001). Indeed, cut-off values of > 30%, > 40%, and > 50% also were correlated significantly with patient outcome, with values of *P* < 0.0001. However, the 10% cut-off value did not reach statistical significance (*P* = 0.075) [23]. The second deficiency was the extensive changes of patients’ clinical stages, because the outcome or survival of cancer patients was affected by their clinical stages to a great degree. Indeed, according to our results, patients with stage I–III who had high/positive cyclin B1 expression had poor 3 or 5-year OS. Besides, we also found that distant metastasis was significantly associated with positive/high expression of cyclin B1, but to combine the results, we regarded stage 0–III as no metastasis and IV as distant metastasis in study of Murakami H et al. 1999 [25]. In addition, in the study of Nozoe T et al. 2002 [24], because of only 1, 3 and 5-year survival rate were given, we converted them to the survival number of patients to make the pooled analysis, which may lead to some bias. Finally, the prognosis were also affected by many of other composite factors, such as tumor histological type, accepting adjuvant therapy or not, patients age, tumor size and venous involvement or not, which should also be taken into consideration.

Considering the limitations above, further research with different clinical stages should be designed to clear the association between positive/high expression of cyclin B1 and prognosis of patients. In addition, further studies should demonstrate which one should be regarded as the optimal cut-off value of cyclin B1 expression for predicting the prognosis of patients with digestive cancer.

In conclusion, according to the present analysis, we could cautiously come to the conclusion that positive/high expression of cyclin B1 may associate with poor prognosis in patients with esophageal cancer, but better prognosis in patients with colorectal cancer, with consideration of the evident statistical significance. In addition, cyclin B1 expression may be associated with size of tumor and distant metastasis. Elevated cyclin B1 expression may be used as one of prognostic indicators for early identification of poor prognosis for patients with digestive cancer.

## MATERIALS AND METHODS

### Criteria for considering studies of this meta-analysis

The including criteria: (1) Immumohistochemical staining of cyclin B1 with surgical specimens; (2) Included people with a pathological diagnosis of EC, GC or CRC; (3) All of randomized, controlled trials (RCTs), observational prospective or retrospective studies were included; (4) Definition of positive/high expression of cyclin B1 was reported; (5) The outcomes including overall survival was reported; (6) Sufficient data (number of cyclin B1 positive patients and their characteristics) were provided.

Excluding criteria: (1) Studies about animals; (2) Non-research studies such as expert opinions, letters, editorials, reviews and case reports; (3) Studies whose participants complicated with other primary tumors; (4) Studies without sufficient data; (5) The definition of positive/high expression of cyclin B1 (cut-off value) was not given or did not meet our including criteria.

### Types of objective measures

The primary objective was the association between cyclin B1 high/positive expression and 3-year OS or 5-year OS.

The secondary objective was the association between cyclin B1 high/positive expression and clinical features including Age, Gender, Size of tumor, Histologic type, Pathologic T, Pathologic N, distant metastasis, Blood vessel invasion, Lymphatic vessel invasion and clinical TNM stage.

### Search strategy

Database of PubMed, EMBASE, Cochrane Library and Web of Science were searched online to January, 2017. Our searching terms and procedures were as follows: (1)“cyclin B1”; (2) “esophageal cancer” OR “esophageal carcinoma” OR “esophageal tumor”; (3) “gastric cancer” OR “gastric carcinoma” OR “gastric tumor”; (4) “colorectal cancer” OR “colorectal carcinoma” OR “colorectal tumor”; (5) “prognosis” OR “survival” OR “outcome”. Search strategy was: 1 AND (2 OR 3 OR 4) AND 5. Two researchers independently screened the titles and abstracts of each study. Then full text will be obtained for further evaluation if one get our including criteria.

### Quality assessment

We used the 9-star Newcastle-Ottawa Scale (NOS) [38] to assess the quality of all included studies, and the total scores of each study were displayed in the characteristics table (Table 1). Three aspects of NOS of evaluation, including selection, comparability, and outcome between the case group and control group were offered for scoring. We consider studies with the scores ≥ 6 as high-quality.

### Data extraction

Data for the analysis were extracted independently by two reviewers. When there was disagreement during the selection process, it was resolved by discussion. The extracted contents including study demographics, published years, country, tumor location, treatment, follow-up time, definition of positive/high expression, age, gender, size of tumor, histologic type, pathologic T, pathologic N, distant metastasis, blood vessel invasion, lymphatic vessel invasion and clinical stage were extracted using a standardized form. Data collected were input into RevMan 5.2 software for analysis [39].

### Statistical analysis

In the present analysis, the prognostic value of cyclin B1 expression in patients with digestive cancer was measured by estimating the Odds Ratio (OR) between high/positive expression of cyclin B1 groups and low/negative expression of cyclin B1 groups. The associated 95% Confidence Intervals (CI) were also measured. The heterogeneity between studies was evaluated with *P* value and *I^2^*. *I^2^* ≥ 50% or *P ≤* 0.10 was deemed to represent significant heterogeneity [40, 41], and pooled OR was estimated using a Random-effect model. On the contrary, if statistical study heterogeneity was not observed (I^2^ ≤ 50% and *P* ≥ 0.10), a fixed effects model was used. The association between the expression of cyclin B1 and clinical characteristics of patients was evaluated in the same way. For the pooled OR estimates of OS, we performed subgroup analysis by Clinical stage (I–IV vs. I–III), *P* value of TNM stage (*P* < 0.05 vs. *P* > 0.05) and Cut-off value (10% vs. ≤ 5%). Besides, we performed sensitivity analysis to examine the stability of the combined results and to identify the source of heterogeneity. Finally, Begg's and Egger's test were used to assess the publication bias. No obvious publication bias was detected, if the shape of funnel plots revealed no obvious asymmetry. All statistical analyses were performed using standard statistical procedures provided in RevMan 5.2 [39].
